# Quantitative electroencephalography predicts postoperative delirium in cardiac surgical patients after cardiopulmonary bypass: a prospective observational study

**DOI:** 10.3389/fmed.2023.1163247

**Published:** 2023-10-27

**Authors:** Yuechuan Xue, Wanglin Liu, Longxiang Su, Huaiwu He, Huan Chen, Yun Long

**Affiliations:** Department of Critical Care Medicine, Peking Union Medical College Hospital, Chinese Academy of Medical Science, Beijing, China

**Keywords:** postoperative delirium, quantitative electroencephalography, critical care, cardiopulmonary bypass, delirium biomarkers

## Abstract

**Objective:**

Despite its frequency and associated negative effect, delirium remains poorly recognized in postoperative patients after ICU admission, especially among those who have undergone cardiac surgery with cardiopulmonary bypass. Postoperative delirium is triggered by a wide variety of acute medical conditions associated with impaired neuronal network connectivity. The lack of objective biomarkers primarily hinders the early detection of delirium. Seeking early biomarkers for tracking POD could potentially assist in predicting the onset of delirium and assessing the severity of delirium and response to interventions.

**Methods:**

QEEGs were taken from 46 sedated postoperative patients, with 24 of them having undergone cardiac surgery. The assessment of delirium was performed twice daily using the Confusion Assessment Method for the ICU (CAM-ICU) to screen for postoperative delirium (POD). QEEG data were interpreted clinically by neurophysiologists and processed by open-source EEGLAB to identify features in patients who had or did not have POD after cardiac or non-cardiac surgery.

**Results:**

The incidence of delirium in patients after undergoing cardiac surgery was nine times greater than in those after non-cardiac surgeries (41.7% vs. 4.5%; *p =* 0.0046). Patients with delirium experienced longer use of mechanical ventilation (118 h (78,323) compared to 20 h (18,23); *p <* 0.0001) and an extended ICU length of stay (7 days (6, 20) vs. 2 days (2, 4); *p <* 0.0001). The depth of anesthesia, as measured by RASS scores (*p =* 0.3114) and spectral entropy (*p =* 0.1504), showed no significant difference. However, notable differences were observed between delirious and non-delirious patients in terms of the amplitude-integrated EEG (aEEG) upper limit, the relative power of the delta band, and spectral edge frequency 95 (SEF95) (*p =* 0.0464, *p =* 0.0417, *p =* 0.0337, respectively).

**Conclusion:**

In a homogenous population of sedated postoperative patients, robust qEEG parameters strongly correlate with delirium and could serve as valuable biomarkers for early detection of delirium and assist in clinical decision-making.

## Introduction

Delirium is a common acute brain dysfunction characterized by fluctuating mental status with temporal and spatial disorientation, inattention, and disorganized thinking. It is associated with negative outcomes, including prolonged intensive care unit (ICU) and hospital stays, increased days of mechanical ventilation, and both short-term and long-term cognitive decline, leading to higher mortality rates (1). Postoperative delirium (POD) is a form of delirium commonly observed during postoperative periods and following anesthesia, especially in patients who have undergone cardiac surgery involving cardiopulmonary bypass in the ICU. The incidence of POD in older patients is estimated at 4.1–54.9% after cardiac and non-cardiac surgery (2–5). The duration of delirium is an independent risk factor for cognitive impairment in critically ill patients (6). Cardiac surgery after cardiopulmonary bypass carries a higher risk of delirium, and cardiopulmonary bypass is implicated in the etiology of delirium. Multiple etiological factors may contribute to delirium in a patient. Regardless of the primary etiology, impaired neuronal network connectivity that becomes less integrated may be the final driver of delirium syndrome (7). However, no quantitative biomarkers could track neural network disturbance (8). It is poorly understood that the contributor to POD involves qEEG features in cardiac surgery patients requiring cardiopulmonary bypass. Therefore, seeking early biomarkers for tracking POD could potentially assist in the advanced detection, diagnosis, and clinical management of delirium.

Electroencephalography (EEG) is a handy and highly sophisticated clinical brain monitoring tool. It provides a non-invasive bedside means to assess the function of cortical and subcortical neural networks. Nevertheless, EEG relies on trained electroencephalographers to interpret EEG features, limiting its use in ICU (9). Quantitative EEG (qEEG) breaks the barriers and easily integrates large amounts of raw critical care continuous EEG (cEEG) data. It is widely used in patients with seizures and ischemia, for vasospasm detection, for identifying the depth of sedation, and for obtaining the prognosis after cardiac arrest (10). Postoperative delirium is triggered by a wide variety of acute medical conditions. Neural network disturbance is a likely mechanistic linker between neuroinflammation and cognitive impairments in psychiatric disorders (11). EEG is greatly sensitive to cortical and subcortical neuronal potential changes. However, a poorly understood contributor to postoperative delirium involves EEG features in critically ill patients (12). EEG is not diagnostic yet. Delta or theta waves are generated by the thalamus and cells in layers II-VI of the cortex, while alpha waves derive from cells in layers IV and V of the cortex (13). The decline in the Alpha-Delta ratio (ADR) indicates decreased connectivity strength and decreased network integration. The combination of EEG wave morphology and qEEG could append and quantify neuronal electrical activity associated with the fluctuating disturbance of consciousness and serve as a potential solution to detect delirium onset and its severity (14). Understanding POD EEG patterns and qEEG parameters is the precondition and the key to its prediction and early interventions for cardiac and non-cardiac postoperative delirium.

This study aimed to develop a qEEG monitoring method to distinguish patients with delirium after non-cardiac surgery from those after cardiac surgery requiring cardiopulmonary bypass. We plan to derive qEEG characteristics from discriminating delirium from non-delirium under sedation, focusing on effectively implementing delirium detection and prevention strategies.

## Method

### Study design and patients

This study was a prospective observational study conducted on postoperative patients receiving non-cardiac and cardiac surgery in the ICU of Peking Union Medical College Hospital (PUMCH). The inclusion criteria were patients scheduled for non-cardiac and cardiac surgery requiring cardiopulmonary bypass (e.g., coronary artery bypass grafting, heart valve repair/replacement, and aortic repair/replacement) with postoperative admission to the ICU for at least 24 h from January 2022 to September 2022. Cardiopulmonary bypass is implicated in the etiology of delirium. We plan to find the mechanism by which cardiopulmonary bypass precipitates delirium using quantitative EEG. It is poorly understood that the contributor to postoperative delirium involves qEEG features in cardiac surgery patients requiring cardiopulmonary bypass. Non-cardiac surgery patients served as the control group without cardiopulmonary bypass circuits. Both cardiac and non-cardiac surgeries (e.g., cytoreductive surgery and pancreatoduodenectomy) were complex (Level 4 surgery) and performed by experts with many years of experience. EEG data were collected under sedation within 24 h of the patients’ admission to the ICU after the surgery. Delirium was assessed twice a day during the ICU stay.

**Figure 1:**
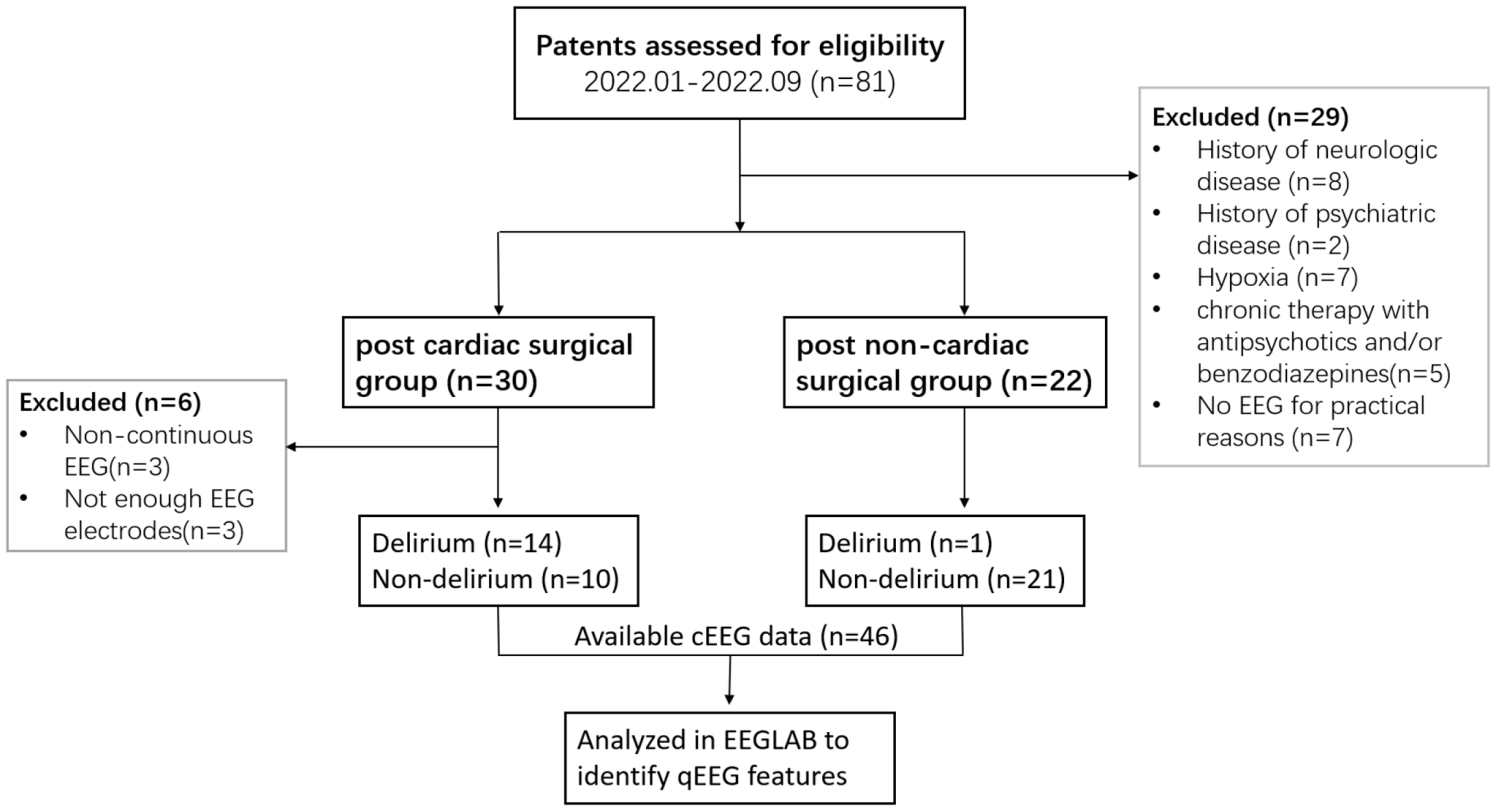
A flow diagram for the inclusion and exclusion of eligible patients.

The exclusion criteria were severe neurologic and psychiatric disease, hypoxia, hypoglycemia, life-threatening organic conditions, chronic therapy with antipsychotics and/or benzodiazepines, blindness, deafness, and an inability to speak Chinese. Patients were matched on age, gender, and comorbid conditions. This prospective observational study was approved by the Institutional Research and Ethics Committee (IREC) of PUMCH (number JS-3575). Patients are free to consent or withdraw from this non-invasive, harmless study. Eligible participants will sign the written informed consent before enrollment. A flowchart for the inclusion and exclusion of eligible patients is shown in Figure 1.

### Postoperative delirium diagnosis

The gold standard for delirium is psychiatric assessment according to DSM-5 criteria, but it requires systematic psychiatric training. For ICU patients, the combination of the Richmond Agitation and Sedation Scale (RASS) (15) and the Confusion Assessment Method for the ICU (CAM-ICU) (16) is an alternative, efficient, and validated delirium assessment. CAM-ICU has excellent agreement with DSM-5 criteria for delirium. It was performed twice a day to screen for POD from four essential aspects: 1) acute onset, 2) altered level of consciousness, 3) inattention, and 4) disorganized thinking, with a sensitivity of 94% and a specificity of 89%. POD incidence was coded as a binary variable defined by CAM-ICU, positive or negative.

### EEG data collection

All EEG acquisitions were performed on a Nicolet EEG instrument with amplifiers controlled by Nicolet™ EEG system software (Natus Neurology Incorporated, Middleton, WI, United States). A wearable headset device was utilized for all postoperative patients to obtain consistent and adequate-quality EEG data. It can adjust its circumference and electrode position according to the patient’s head features for a proper fit. The participants wore the device for 1 h for EEG recording under sedation within 24 h after they were admitted to the ICU. RASS scores and EEG spectral entropy were used to assess the depth of anesthesia. Most postoperative patients were in moderate or deep sedation (RASS−3 or SE 40–50), which means that they could not complete any instructions, and no agitation was observed during this process. EEG acquisition was conducted in accordance with the international 10/20 system, using Fz as the ground electrode and Cz as the reference electrode. All electrode impedances were confirmed to be lower than 10 KOhm. We set the sampling rate to 256 Hz and adjusted the band-pass filter from 0.1 to 40 Hz. We then configured the timebase to 30 mm/s and the sensitivity to 70 uV/cm before initiating the recording.

### QEEG processing and analyzes

EEG data were analyzed to derive qEEG characteristic parameters in the longitudinal bipolar montage using open-source EEGLAB (version 2022.0; https://eeglab.org/) processing in MATLAB 2022 (Mathworks, MA, United States). Raw EEG data were filtered by a 0.5 Hz high-pass and a 40 Hz low-pass filter. In our study, qEEG-derived variables are listed in Table 1. Spectral entropy could objectively reflect the brain’s electric behavior in response to the depth of anesthesia through the complexity of EEG signals. Amplitude-integrated EEG was defined as the lower and higher border amplitudes of the EEG signal according to cerebral function and integrated brain activity. The relative alpha variability was defined uniquely as 6–14 Hz frequency power divided by the total power (1–20 Hz) over a 2-min recording, reflecting cerebral blood flow. Relative spectral power was computed using a fast Fourier transformation and averaged over all channels and epochs. QEEG data were carefully interpreted with raw EEG data and double-checked by neurophysiologists, excluding the interference of the ICU environment. EEG quantitative parameters were presented as the average value of the left and right frontal (Fp1, Fp2), central (C3, C4), temporal (T3, T4), and occipital (O1, O2) EEG recording channels.

**Table 1:** EEG quantitative parameters involved in this study.

Descriptive	Explanation and clinical application
Spectral Entropy (SE)	Monitor the depth of anesthesia and titrate anesthetic agents
Amplitude-integrated EEG (aEEG)	Monitor cerebral function and integrate brain activity
Relative Alpha Variability (RAV)	Reflect cerebral blood flow
Relative Power Per Frequency Band	Relative Power of delta, theta, alpha, and beta bands
Spectral Edge Frequency 95 (SEF95)	EEG frequency below 95% of the spectrogram

### Sample size and power

Based on the patient database of PUMCH in the past 5 years, the POD incidence of cardiac and non-cardiac groups is 35.3% versus 4.6%. Thus, we assume that the incidence of POD would be 35% in the cardiac cardiopulmonary bypass surgery group and 5% in the non-cardiac surgery group. The clinical difference in POD incidence in the two groups would be 30%. Setting two-tailed type I error 0.05 and statistical power 0.80, *n* = 21 patients per group, would enable us to detect that absolute difference. Therefore, we planned to recruit 21 participants undergoing elective surgery in each group at PUMCH.

### Statistical analysis

The qEEG characteristic parameters were presented as means with standard deviations (SD). Pairwise comparisons between the non-cardiac and cardiac groups were investigated using Welch’s *t*-test (two-sided) or a one-way analysis of variance (ANOVA). Statistical analysis was carried out using SPSS version 26 (IBM, Armonk, NY, United States).

## Result

### Patient characteristics and outcome

A total of 46 patients were involved in this study, of whom 24 were admitted to the ICU after cardiopulmonary bypass surgery. Patient characteristics are shown in Table 2. Ten of 24 cardiac surgical patients were delirious, and 14 were not delirious. POD incidence was significantly higher in those who had undergone cardiac surgery [41.7% vs. 4.5%; *p* = 0.0046]. Patients with delirium had longer hours of mechanical ventilation [118 h (78,323) vs. 20 h (18,23); *p <* 0.0001] and ICU length of stay [7 (6, 20) vs. 2 (2, 4); *p <* 0.0001] after cardiac surgery. The baseline scores of APACHE II and SOFA in the delirious and non-delirious groups were 14 (13,17) vs. 12 (11,15) [*p =* 0.0746] and 10 (8, 13) vs. 6 (5, 8) [*p <* 0.0001], respectively. Despite significantly different doses of midazolam used between groups, there was no difference in the depth of anesthesia assessed by RASS scores or spectral entropy. Therefore, to minimize the disturbance of sedation after ICU admission, we consistently conducted and recorded study-specific EEGs both between and within groups.

**Table 2:** Patient characteristics and outcome.

Descriptive	Cardiac surgery (*n* = 24)		Non-cardiac Surgery (*n* = 22)	*p* value
	Delirium (*n* = 10)	Non-delirium (*n* = 14)		
Age, mean (SD), year	64 (8)	58 (15)	65 (11)	0.2299
Gender, male sex	8 (80)	12 (86)	18 (81)	0.9273
SOFA Score	12 (11, 13)	8 (7, 10)	6 (5, 8)	<0.0001^****^
APACHE II	16 (14, 19)	13 (13, 15)	12 (11, 15)	0.0782
Medication administration				
Propofol	10 (1ACT00)	14 (100)	22 (100)	1.0000
Midazolam	8 (80)	12 (86)	1 (4.5)	<0.0001^****^
Fentanyl	10 (100)	14 (100)	22 (100)	1.0000
Depth of anesthesia				
RASS	−3 (−3.8, −3)	−3 (−3, −3)	−2.5 (−3, −2.5)	0.3114
SE, mean (SD)	38.1 (4.1)	39.9 (6.0)	42.9 (7.5)	0.1504
Delirium	10 (41.7)		1 (4.5)	0.0046^**^
Mechanical Ventilation, hour	118 (78, 323)	20 (18, 23)	5 (6, 15)	<0.0001^****^
ICU length of stay, day	7 (6, 20)	2 (2, 4)	1 (1, 1)	<0.0001^****^
Hospital mortality	1 (10.0)	0 (0.0)	0 (0.0)	0.1588

Data are presented as a number (%) or median (IQR) hour unless otherwise indicated. SOFA, Sequential Organ-Failure Assessment; APACHE II, Acute Physiology and Chronic Health Evaluation II; RASS, Richmond Agitation-Sedation Scale; SE, Spectral Entropy; IQR interquartile range; ICU intensive care unit; *Significant difference at two-sided *t*-test with a *p*-value of <0.05 (*p < 0.05, **p < 0.01, ***p < 0.001, ****p < 0.0001).

### Quantitative EEG analysis

**Figure 2:**
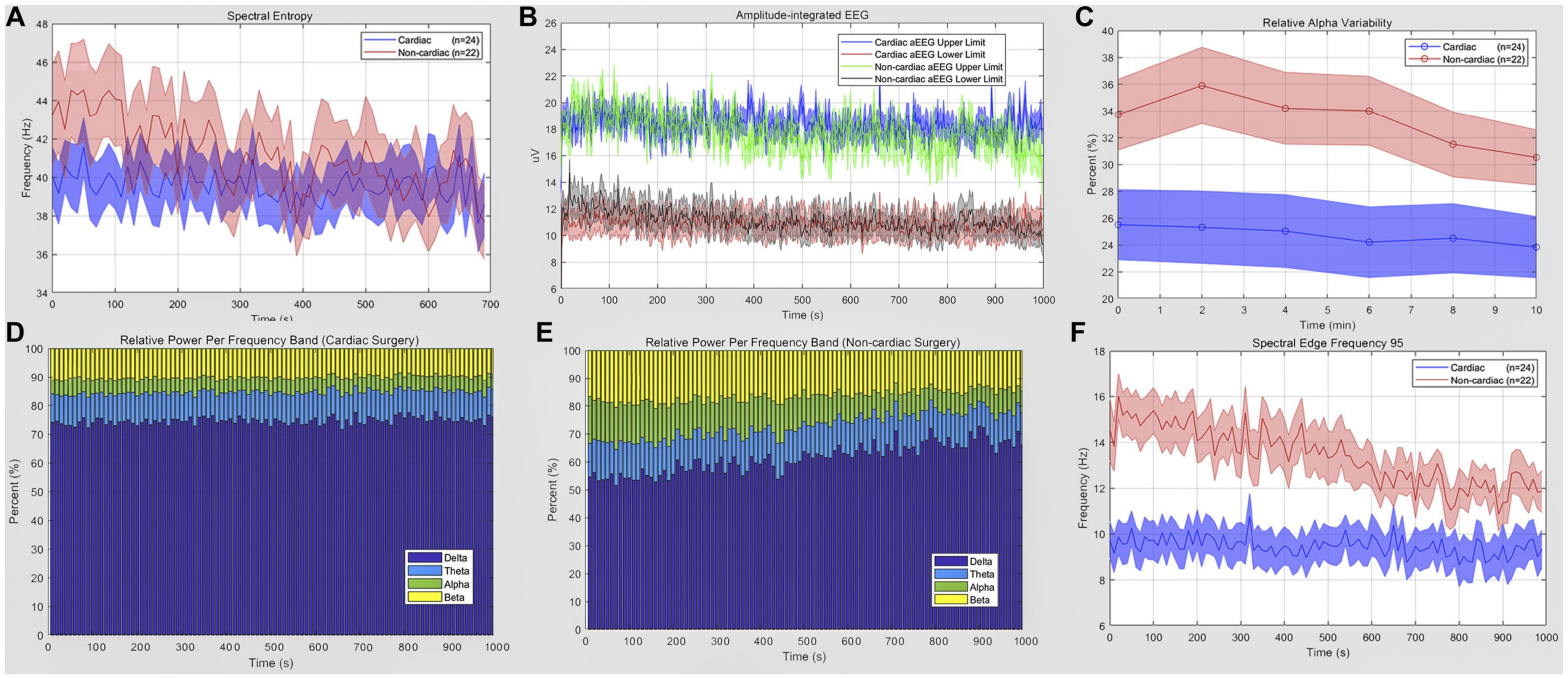
Quantitative EEG analysis of cardiac and non-cardiac surgical patients. **(A)** Spectral entropy estimates for the cardiac (blue) and non-cardiac (red) groups. **(B)** Amplitude-integrated EEG of two groups; data are presented as upper and lower limit; blue and red for cardiac subjects; green and gray for the other. **(C)** Relative alpha variability for cardiac (blue) and non-cardiac (red) subjects. **(D,E)** Relative power per frequency band of delta (purple), theta (blue), alpha (green), beta (yellow) wave for patients. **(F)** Lower spectral edge frequency 95 in the cardiac group (blue) compared to the control(red) (*p* = 0.0003). Data are presented as mean ± SEM for the average value of all eight recoding channels and epochs.

POD mainly occurred in the first 48 h (8 of 11) after ICU admission. The mean (standard error of mean, SEM) of qEEG characteristics has been displayed in Figure 2. Table 3 shows the quantitative analysis of EEG derivations between the cardiac and non-cardiac groups. For the variables identified, there was no statistically significant difference in spectral entropy (SE) and amplitude-integrated EEG (aEEG) upper and lower limits between groups (*p* = 0.0885, *p* = 0.4580, *p* = 0.3079, respectively) in this study. The mean (SEM) relative alpha variability (RAV) for each group was 24.9 (2.4) for the cardiac group and 33.4 (2.78) for the non-cardiac group (*p* = 0.0295). The relative delta wave increased (*p* = 0.0006), while the alpha (*p* = 0.0056) and beta (*p* = 0.0062) waves decreased in cardiac surgical patients. This corresponded to a lower spectral edge frequency of 95 (SEF95) (9.46 vs. 14.31 Hz; *p* = 0.0003).

**Table 3:** QEEG characteristics.

qEEG Descriptive	Cardiac surgical patients (*n* = 24)	Non-cardiac surgery patients (*n* = 22)	*p* value
Spectral Entropy (SE)	39.29 ± 1.32	42.89 ± 1.57	0.0885
Amplitude-integrated EEG (aEEG)			
Upper Limit, uV	15.01 ± 1.09	16.38 ± 1.54	0.4580
Lower Limit, uV	9.92 ± 0.66	11.11 ± 1.01	0.3079
Relative Alpha Variability (RAV), %	24.92 ± 2.431	33.38 ± 2.772	0.0295*
Relative power per frequency band			
Delta Wave, %	74.95 ± 2.32	58.49 ± 4.15	0.0006***
Theta Wave, %	9.85 ± 0.76	11.64 ± 0.78	0.1291
Alpha Wave, %	10.23 ± 1.49	17.27 ± 1.90	0.0056**
Beta Wave, %	4.97 ± 0.77	12.61 ± 2.35	0.0062**
Spectral Edge Frequency 95 (SEF95)	9.46 ± 0.75	14.31 ± 1.01	0.0003***

Data are presented as mean ± SEM. An unpaired t-test or Welch’s unequal variances t-test was used to test the difference in quantitative EEG parameters. RAV, the relative power of delta, alpha, beta band, and SEF 95 showed a statistically significant difference between cardiac and non-cardiac surgical patients (*p < 0.05, **p < 0.01, ***p < 0.001, ****p < 0.0001).

**Figure 3:**
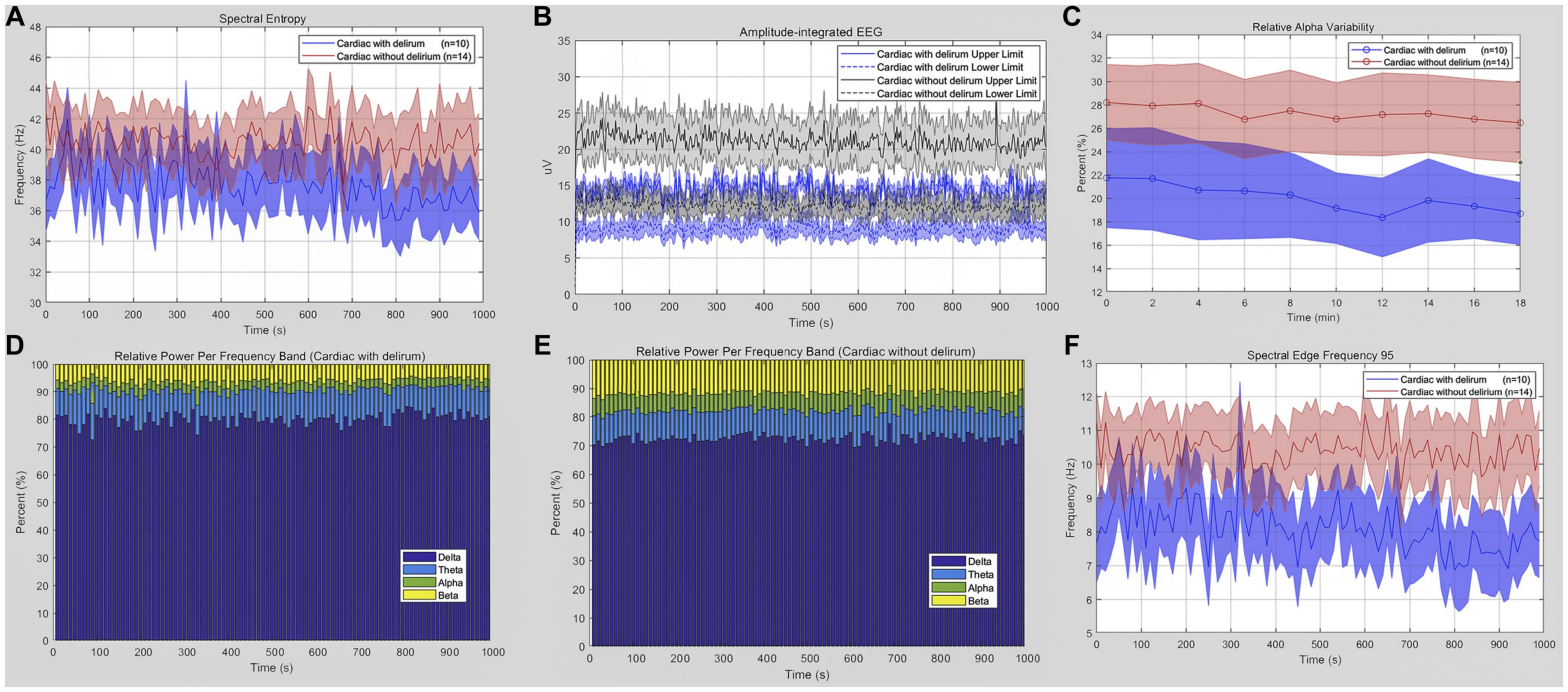
Quantitative EEG analysis of delirious or non-delirious surgical patients. **(A)** Spectral entropy estimates for delirious (blue) and non-delirious (red) subjects. **(B)** Amplitude-integrated EEG upper and lower limit (delirium: blue; non-delirium: gray). **(C)** Relative alpha variability for delirious (blue) and non-delirious (red) subjects. **(D,E)** Relative power per frequency band of delta (purple), theta (blue), alpha (green), and beta (yellow) waves for patients. The relative delta wave increased (*p* = 0.0417), while the alpha and beta waves decreased in delirium patients. **(F)** Lower spectral edge frequency 95 in delirium patients (blue) compared to the control (red) (*p* = 0.0337). Data are presented as mean ± SEM for the average value of all eight recording channels and epochs.

When we restricted our analyzes to intra-group cardiac surgical patients, we compared qEEG data in post-cardiac patients with delirium and those without delirium. Figure 3 shows the qEEG characteristic trend between the delirious and non-delirious groups. The upper limit of aEEG, the relative power of the delta band, and SEF 95 showed a statistically significant difference (*p* = 0.0464, *p* = 0.0417, *p* = 0.0337, respectively). At the same time, there was no significant difference in SE, RAV, or the relative power of the theta, alpha, and beta bands (Table 4). The unpaired t-test, or Welch’s unequal variances t-test, indicated a substantial reduction in the EEG upper limit, SEF 95, and an increment in delta power in delirious subjects across all EEG recording channels and epochs.

**Table 4:** qEEG characteristics of cardiac surgical patients with/without delirium.

qEEG Descriptive	Cardiac surgical patients with delirium (*n* = 10)	Cardiac Surgical Patients without Delirium (*n* = 14)	*p* value
Spectral Entropy (SE)	38.14 ± 1.33	39.94 ± 1.91	0.4907
Amplitude-integrated EEG (aEEG)			
Upper Limit, uV	12.16 ± 1.321	16.66 ± 1.531	0.0464*
Lower Limit, uV	8.23 ± 0.84	10.84 ± 0.92	0.0574
Relative Alpha Variability (RAV), %	22.24 ± 3.57	26.77 ± 3.31	0.3803
Relative power per frequency band			
Delta Wave, %	80.29 ± 1.62	71.02 ± 3.342	0.0417*
Theta Wave, %	9.85 ± 0.56	10.87 ± 1.12	0.4663
Alpha Wave, %	6.42 ± 1.26	11.88 ± 2.15	0.0657
Beta Wave, %	3.447 ± 0.51	6.24 ± 1.26	0.0945
Spectral Edge Frequency 95 (SEF 95)	7.58 ± 0.81	10.81 ± 1.03	0.0337*

Data are presented as mean ± SEM. An unpaired t-test or Welch’s unequal variances t-test was used to test quantitative EEG parameters’ differences. *Significant difference at two-sided t-test with a *p*-value of *<*0.05. The upper limit of aEEG, the relative power of the delta band, and SEF 95 showed a statistically significant difference between cardiac surgical patients with/without delirium and non-delirium (*p* < 0.05).

## Discussion

The longer a patient suffers from POD, the greater the likelihood of irreversible cognitive impairment and poor quality of life. However, despite the use of continuous delirium screening tools such as CAM-ICU, over 50% of patients with delirium were missed (17). QEEG could potentially provide a non-invasive bedside means to predict POD in the ICU. In this study, POD incidence of cardiac surgical patients was nine times greater than that in the control group (41.7% vs. 4.5%).

Patients with delirium experienced a longer duration of mechanical ventilation use (*p* < 0.0001) and an extended ICU length of stay (*p* < 0.0001). We identified several significant parameters from qEEG as potential biomarkers for the early detection of POD in patients, whether they underwent cardiac or non-cardiac surgery. These findings could assist ICU physicians in implementing an ABCDEF (A2F) bundle (18) to preemptively address POF, even before a clinical diagnosis of POD is made, thereby reducing complications.

To our knowledge, our study is the first to investigate delirious qEEG characteristics under sedation to assist in the advanced detection, diagnosis, and clinical management of delirium. To avoid the interference of sedative medication administration, RASS scores and EEG spectral entropy were used to assess the depth of anesthesia. They were not significantly different in patients who had or did not have POD after cardiac or non-cardiac surgery. Post-cardiac surgical patients received a higher dose of midazolam than post-non-cardiac surgical patients. However, no difference was found in midazolam use between the delirious and non-delirious groups. In the past years, the use of midazolam has stirred controversies about whether it is an independent risk factor for delirium in critically ill patients (6, 18, 19). It probably acted as a precipitating clinical factor, not a predisposing factor. In this study, spectral entropy could objectively reflect the brain’s electric behavior in response to the depth of anesthesia through the complexity of EEG signals (20). Similar spectral entropy made qEEG parameters comparable between and within groups after ICU admission. Most general anesthetics that act primarily by enhancing GABAA receptors could induce burst suppression (21), and the link between burst suppression and POD remains controversial, and studies have contradictory results (22). EEG burst suppression and duration may predict postoperative delirium to some extent and are associated with increased mortality. In our study, qEEG data were carefully interpreted with raw EEG data and double-checked by neurophysiologists. We did not find burst suppression in the raw EEG data.

The first result of this study showed that cardiac surgery carried a higher risk of delirium when compared to other types of surgery. Impaired neuronal network connectivity that becomes less integrated may be the final driver of delirium syndrome. There are currently few known correlations between qEEG and POD. qEEG monitoring revealed several key risk characteristics between groups, such as RAV, relative spectral frequency, and SEF95. Hussein et al. (23) found that RAV can reflect cerebral blood flow (CBF), and its changes precede Alpha-Delta-Ratio (ADR) change. In our study, a decline in the RAV was associated with an increment in alpha and beta power and a reduction in delta power. Delta or theta waves are generated by the thalamus and cells in layers II-VI of the cortex, while alpha waves originate from cells in layers IV and V of the cortex (13). The decline in the Alpha-Delta ratio (ADR) indicates decreased connectivity strength and decreased network integration. To date, it is still not clear whether POD is a direct cause of poor clinical outcomes or a mediating factor affected by other unidentified factors causing delirium and poor outcomes. QEEG parameters may offer an objective tool for the early detection of POD and pave the way for a deeper understanding of its development in future studies.

The other significant finding of our study revealed that delirious patients after cardiac surgery were detectable in qEEG delirium monitoring. Specifically, according to the qEEG data, the aEEG upper limit, generalized delta wave, and SEF 95 were associated with the presence of delirium and its poor clinical outcomes, including prolonged use of mechanical ventilation and an extended ICU stay in cardiac surgical patients after ICU admission. Amplitude-integrated EEG (aEEG) could continuously monitor and integrate brain activity. No seizures or burst suppression were traced in this study by aEEG. The lower and upper limits were more than 5 μV and 10 μV, respectively, within the normal range in cardiac surgical patients. However, delirious patients were less than non-delirious patients in the upper(*p* = 0.0464) and lower limits (*p* = 0.0574). It probably indicated abnormally low brain activity, promoting the development of POD. In previous qEEG studies, relative spectral power was most frequently studied between delirium and non-delirium. Our study is the first to derive multiple qEEG characteristics systematically (SE, aEEG, RAV, relative spectral power, SEF 95) in patients under sedation with/without delirium after cardiac surgery requiring cardiopulmonary bypass.

Another interesting finding of this study was that no sleep–wake cycle occurred in the sedated patients for the whole duration of EEG monitoring, but characteristics with a high proportion (>60%) of slow delta waves were continuously presented. Nevertheless, deep sleep is also characterized by slow-wave activity. Fultz et al. (24) showed that, during sleep, EEG slow-delta waves are coupled with and precede oscillations in cerebral blood flow and CSF within the gray matter. Under physiological conditions, the glymphatic system is mainly regulated by the sleep–wake cycle, and slow-wave sleep (SWS) significantly improves brain waste removal efficiency (25). Future studies should differentiate between sleep–wake rhythmic and persistent SWS when exploring the development of POD. Such insights could inform strategies for the treatment and prevention of postoperative delirium, with guidance from quantitative EEG.

Some limitations of our study should be acknowledged. The limited sample size of delirious patients might introduce bias in the qEEG characteristics of cardiac surgical patients with/without delirium.

The altered arousal states present in three different subtypes of delirium are as follows: hyperactive, hypoactive, and mixed.

Specific qEEG features of each POD phenotype still need to be studied in the future. Notably, hypoactive delirium is the most common ([95% CI, 8–17%]), with an incidence of 11%, in patients admitted to the ICU (26). However, almost all delirious patients (10 of 11) in our study had hyperactive delirium.

Factors such as sample size, the frequency of delirium assessment, and heterogeneity among studies may account for the difference. Moreover, approximately 12% of postoperative patients developed sedation-related delirium after abrupt cessation of sedatives during the spontaneous awakening trial (SAT) within 2 h. In our study, we attempted to differentiate POD from SAT. The patients received sedative medication administration again, reduced it slowly, and then carefully assessed whether it was POD. Owing to an insufficient sample size of delirious patients, the ROC curve was not satisfactory, yielding a sensitivity of 78% and a specificity of 50%. Although a wearable headset allowed ICU physicians to utilize cEEG in practice, the challenging ICU environments still resulted in some residual, unavoidable interference. Furthermore, each patient was monitored only once for baseline qEEG after ICU admission, without subsequent dynamic monitoring in the next few days.

## Conclusion

The primary obstacle to accurate POD diagnosis is the lack of objective biomarkers. While current tools such as CAM-ICU or CDSC are widely used, there is a need for ICUs to adopt an objective, standardized bedside approach for diagnosing POD. Our study underscores the potential of qEEG as a tool for the early detection of POD. The etiology of POD is multifactorial and still needs to be fully elucidated and understood. Therefore, identifying predispositions for POD and understanding EEG characteristics could improve our understanding of POD development.

QEEG monitoring could serve as a non-invasive bedside method to assist in the early detection and clinical management of delirium. In this study, we identified robust qEEG parameters that hold potential in distinguishing and predicting postoperative delirium in cardiac surgical patients after cardiopulmonary bypass.

## Data availability statement

The raw data supporting the conclusions of this article will be made available by the authors, without undue reservation.

## Ethics statement

The studies involving human participants were reviewed and approved by Institutional Research and Ethics Committee (IREC) of PUMCH. The patients/participants provided their written informed consent to participate in this study.

## Author contributions

YX, WL, and LS carried out the experiment. YX and LS analyzed the data. YX and WL wrote the manuscript with support from HH, HC, and YL. YX, HC, and YL conceived the original idea. YL supervised the project. All authors contributed to the article and approved the submitted version.
